# Comparison of the Effects of Long-term Hemodialysis and Peritoneal Dialysis Modalities on Left Ventricular Functions

**DOI:** 10.31083/j.rcm2511401

**Published:** 2024-11-18

**Authors:** Mehmet Usta, Selma Kenar Tiryakioğlu, Alparslan Ersoy, Nur Özer Şensoy, Ömer Furkan Demir, Mustafa Cagatay Buyukuysal

**Affiliations:** ^1^Department of Nephrology, University of Health Sciences, Bursa City Training and Research Hospital, 16140 Bursa, Turkey; ^2^Department of Cardiology, University of Health Sciences, Bursa City Training and Research Hospital, 16140 Bursa, Turkey; ^3^Department of Nephrology, Bursa Uludag University Faculty of Medicine, 16285 Bursa, Turkey; ^4^Department of Internal Medicine, Bursa Uludag University Faculty of Medicine, 16285 Bursa, Turkey; ^5^Department of Cardiology, Bursa Yuksek Ihtisas Training and Research Hospital, 16140 Bursa, Turkey; ^6^Department of Biostatistics, Zonguldak Bulent Ecevit University School of Medicine, 67100 Zonguldak, Turkey

**Keywords:** chronic kidney failure, hemodialysis, peritoneal dialysis, left ventricular hypertrophy, diastolic dysfunction, echocardiography

## Abstract

**Background:**

Hemodialysis (HD) and continuous ambulatory peritoneal dialysis (CAPD) affect left ventricular hemodynamics. This study compared the effect of two treatment modalities, CAPD and HD, on left ventricular systolic and diastolic functions in maintenance dialysis patients.

**Methods:**

A total of 47 patients (24 CAPD and 23 HD) undergoing long-term dialysis were included in the study. Left ventricular functions, left ventricular hypertrophy, and left ventricular geometry were evaluated using echocardiography.

**Results:**

The mean age of the patients was 58.6 ± 11.2 years. The mean dialysis time was 125.1 ± 35.2 months. When echocardiographic parameters were examined, left ventricular muscle mass, mass index, E/e’ ratios, and global longitudinal strain were significantly higher in the CAPD group. The rates of diastolic dysfunction (66.7% *vs*. 26.1%) and left ventricular hypertrophy (91.7% *vs*. 60.9%) were higher in the CAPD group than in the HD group. Dialysis modality CAPD, abnormal global longitudinal strain (GLS), and increased serum calcium were associated with an increased risk of diastolic dysfunction.

**Conclusions:**

The study results demonstrated that left ventricle (LV) diastolic dysfunction and deterioration in left ventricular geometry were significantly higher in patients receiving long-term CAPD treatment than for long-term HD treatment.

## 1. Introduction

Deaths resulting from cardiovascular diseases (CVDs) are considered a significant issue among patients with end-stage kidney disease (ESKD) [1]. Patients with ESKD experience cerebrovascular disease mortality rates that are 20 times greater than those in the general population. Additionally, data from a U.S. database of individuals with kidney failure revealed that cardiovascular diseases account for roughly 39% of deaths among dialysis patients [1, 2]. Left ventricular hypertrophy (LVH), which is not uncommon in patients with chronic kidney disease (CKD), is noted as one of the risk factors for cardiovascular disease and death [2]. Along with LVH, there are also changes in cardiac structure and function, which are shown to be prognostic factors in ESKD patients receiving hemodialysis (HD) treatment [3]. Cardiac abnormalities in these patients may develop secondary to multiple factors, including chronic volume and pressure overload, anemia, uremia, high-flow arteriovenous shunts, abnormal calcium and phosphate metabolism, and hyperparathyroidism [4].

LVH is the most common cardiovascular abnormality in patients with CKD [5]. The prevalence of LVH in non-dialysis-dependent CKD patients is around 47%. In comparison, the prevalence of LVH among patients treated with HD or continuous ambulatory peritoneal dialysis (CAPD) is reported to be approximately 75% [6]. When patients who developed LVH were examined, continuing dialysis treatment or the type of dialysis continued did not cause LVH to regress significantly [7].

Although adverse effects of chronic dialysis treatments on left ventricular geometry had be shown, the results of short- and multiple-year cross-sectional studies comparing HD and CAPD treatments on the left ventricle functions were controversial [8]. Hence, we sought to compare the association of dialysis treatment on left ventricular systolic and diastolic functions and left ventricular geometry in HD and CAPD patients.

## 2. Materials and Methods

### 2.1 Data Collection and Laboratory Analysis

This prospective, observational, and cohort study included patients receiving dialysis treatment for at least 7 years in a tertiary care center between June 2020 and January 2023. Exclusion criteria for this study included: (i) a dialysis duration less than 7 years, (ii) previously diagnosed coronary artery disease, (iii) rhythm and conduction abnormalities, (iv) heart valve diseases, (v) thyroid dysfunction, (vi) chronic obstructive pulmonary disease, (vii) rheumatic diseases, (viii) previously diagnosed heart failure, (ix) active malignancy or active infection, (x) switching between dialysis methods, (xi) patients with missing data, and (xii) patients lost during the follow-up. The inclusion criteria for this study were defined as (i) having received dialysis treatment for more than 7 years and (ii) being suitable for detailed examination of echocardiographic images.

**Fig. 1.**
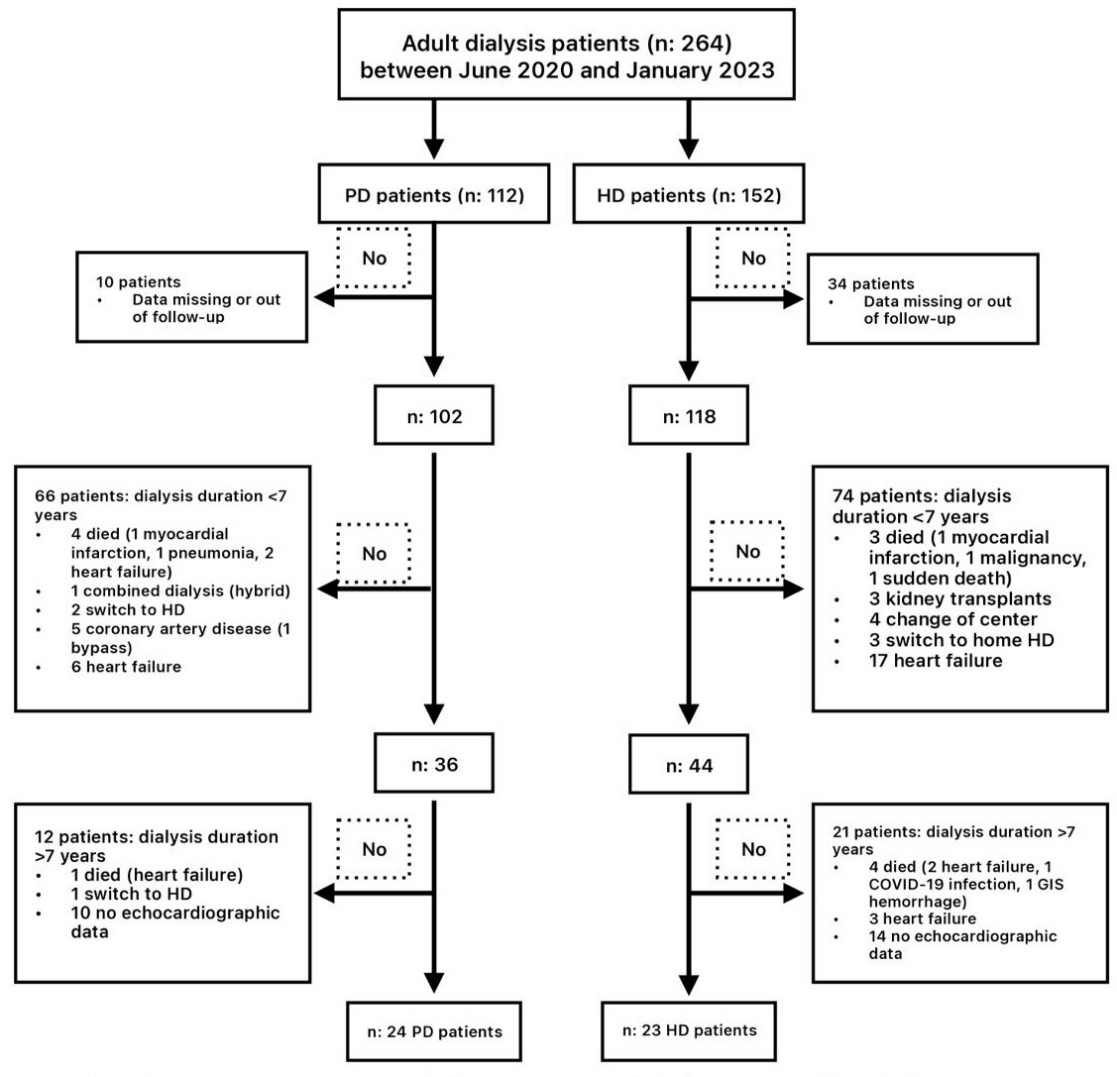
**Flowchart of the study**. Abbrevations: COVID-19, corona virus disease 2019; GIS, gastrointestinal system; HD hemodialysis; PD, peritoneal dialysis.

After patients were excluded according to the existing criteria, the study continued with 47 patients. In total, 23 of these patients received HD, and 24 received CAPD treatment. A flowchart of the patients included in the study is shown in Fig. 1. Patients were followed for an average of 6 months after echocardiographic evaluation. The secondary aim of this study was to determine the frequency of deaths and hospitalization during the 6-month follow-up period.

Patients included in the study were questioned clinically for heart failure symptoms. The clinical condition, medical history, physical examinations, and imaging tests (electrocardiyography, chest X-ray) of each patient were examined for signs of heart failure. According to the universal heart failure definition, heart failure is a clinical syndrome characterized by symptoms and/or signs arising from structural or functional cardiac abnormalities and/or objective evidence of pulmonary or systemic congestion [9].

Body mass index (BMI (kg/m^2^) = body weight (kg)/height (m^2^)) and body surface area (BSA (m^2^) = √ height (cm) × weight (kg)/3600) were calculated [10]. Blood samples were collected from patients receiving HD treatment before the first dialysis session and from CAPD patients after a long interval before dialysis following overnight fasting. Complete blood count and extensive patient biochemistry blood samples were sent to the laboratory as appropriate.

### 2.2 Dialysis Characteristics

Dialysis vascular access was with a permanent dialysis catheter in 1 patient and an arteriovenous fistula (AVF) in 22 patients. Only 1 of the 22 HD patients had the AVF in a brachiocephalic location. All HD patients received standard bicarbonate (in mmol/L; bicarbonate: 32, acetate: 3, Na+: 140, K+: 2, ionized Ca++: 1.5, Mg++: 0.5, chloride: 111) dialysis treatment was performed thrice weekly using high-flux dialyzers (FX 80, ultrafiltration coefficient 59 mL/h × mmHg, effective surface 1.8 m^2^, priming volume 95 mL, membrane material Helixone®, housing material polypropylene, potting compound polyurethane and sterilization method INLINE Steam, Fresenius Medical Care, Bad Homburg, Germany) and the Fresenius 4008 B device (Fresenius Medical Care, Bad Homburg v.d. Höhe, Germany). The ultrafiltration volume (mL/kg/hour) was adjusted in each dialysis session after considering the hemodynamics and volume status of each patient. A reverse osmosis purification system (Aqua RO modular, Fresenius Medical Care, Bad Hamburg, Germany) with an endotoxin filter was employed to offer dialysis water for the single-use hemodialyzers.

Except for the two patients who underwent automated peritoneal dialysis (APD), all CAPD patients received manual exchanges four times daily. Seven patients used Dianeal® (Baxter, Baxter,Unterschleißheim, Germany) peritoneal dialysis solution, and 16 used Stay-Safe Balance® (Fresenius, Fresenius, Bad Homburg v.d. Höhe, Germany) solution. A standard peritoneal equilibration test was used to evaluate the transport characteristics of the peritoneal membrane. Standard fluid and dietary restrictions (1.2 g/kg/day protein, 50 mmol sodium, restricted potassium, and phosphate) were applied to all patients.

Anuria was defined by a urine output of under 100 mL per day. Residual renal function was not estimated if the 24-hour urine output was below 100 mL. Dialysis adequacy was traditionally assessed using the urea reduction ratio (URR) and Kt/Vurea, where K represents urea clearance, t signifies dialysis duration, and V denotes the volume of distribution in patients, based on pre- and post-dialysis concentrations. The following formulas were used to measure Kt/V and URR [11]:

- Daugirdas formula: Kt/Vsp = –ln (R – 0.008 × t) + (4 – 3.5 × R) × Uf/W (ln: natural logarithm, R: ratio of postdialytic ÷ predialytic blood urea nitrogen (BUN), t: effective dialysis time in hours, Uf: ultrafiltration volume in liters, W: weight of the patient after dialysis in kg. Kt/Vsp: single pool Kt/V)
- URR: pre-dialysis urea – post-dialysis urea/pre-dialysis urea

Kt/V_urea_ in CAPD was calculated using the following equation [12]:

- Kt = (D_urea_/P_urea_) × VD (Kt: daily peritoneal urea clearance, D_urea_: urea concentration in pooled drain dialysate (dialysate from all exchanges in 24 hours was pooled, mixed properly, and then the sample was collected to assess D_urea_), P_urea_: plasma urea concentration, VD: 24-hour peritoneal dialysate drain volume).

‘V’ represents the volume of distribution of urea, equivalent to total body water (TBW). Watson’s equation was used to calculate ‘V’ when determining Kt/Vurea for adults whose weight was at or near their dry weight. The equation for males was TBW = 2.447 – (0.09156 × age) + (0.1074 × height) + (0.3362 × weight), and for females: TBW = –2.097 + (0.1069 × height) + (0.2466 × weight). Weekly Kt/V values used for comparison were calculated by obtaining the weekly average of the daily peritoneal dialysis Kt/V urea values and the weekly average of the HD Kt/V values based on the number of HD sessions. The reason for choosing this method is to reflect the differences between the two types of dialysis more accurately and to provide a more meaningful comparison.

### 2.3 Echocardiographic Measurements

Two experienced cardiologists, blinded to the clinical characteristics of the patients, performed the echocardiographic measurements (resting two-dimensional (2D), M-mode, Doppler, and tissue Doppler imaging (TDI)) using the Phillips EpiQ7 device (Andover, MA, USA) and a variable-frequency phased array transducer (2.5–3.5–4.0 MHz). To exclude cardiac effects due to volume load, parasternal long-axis, short-axis, four-chamber, two-chamber, and three-chamber apical images were obtained in the left lateral decubitus position while the dialysis patients were at their dry weight (2 hours after the HD session or CAPD change), and stored digitally (in DICOM format) for offline analysis. All recordings and measurements were averaged over three cardiac cycles, following the echocardiography practice standards. All patients presented a sinus rhythm, and those with atrial fibrillation were excluded from the study.

Left atrium (LA) diameter, end-diastolic interventricular septum (IVSd), end-diastolic posterior wall (PWd), and left ventricle end-diastolic (LVDd) and end-systolic (LVDs) diameters were recorded. Left ventricular mass and left ventricular mass index (LVMI) were calculated using the formulas recommended by the American Society of Echocardiography and indexed to BSA as follows [13]:

- left ventricular mass = 0.8 × (1.04 × ((LVDd + PWd + IVSd)^3^ – (LVDd)^3^)) + 0.6
- LVMI = left ventricular mass/BSA

LVH was defined as an increased LVMI greater than 95 g/m^2^ in women and an increased LVMI greater than 115 g/m^2^ in men. LVH categories were divided into four for male and female patients, respectively, according to sex-specific cutoffs, as recommended: no LVH (<116 g/m^2^ and <96 g/m^2^); mild LVH (≥116 to <132 g/m^2^ and ≥96 to <109 g/m^2^); moderate LVH (≥132 to ≤148 g/m^2^ and ≥109 to ≤121 g/m^2^); severe LVH (>148 g/m^2^ and >121 g/m^2^) [14, 15]. Relative wall thickness (RWT) was calculated using the formula (2 × PWd)/(LVDd) [16]. The geometric changes in the left ventricle were classified based on LVMI and RWT. Four distinct groups were identified: elevated RWT (>0.42) combined with increased LVMI (>115 g/m^2^ for men and >95 g/m^2^ for women) was classified as concentric hypertrophy, elevated RWT (>0.42) with normal LVMI (≤115 g/m^2^ for men and ≤95 g/m^2^ for women) was termed concentric remodeling, normal RWT (≤0.42) with increased LVMI was labeled eccentric remodeling, and normal left ventricle (LV) geometry was defined by having both normal RWT and LVMI [16].

**Fig. 2.**
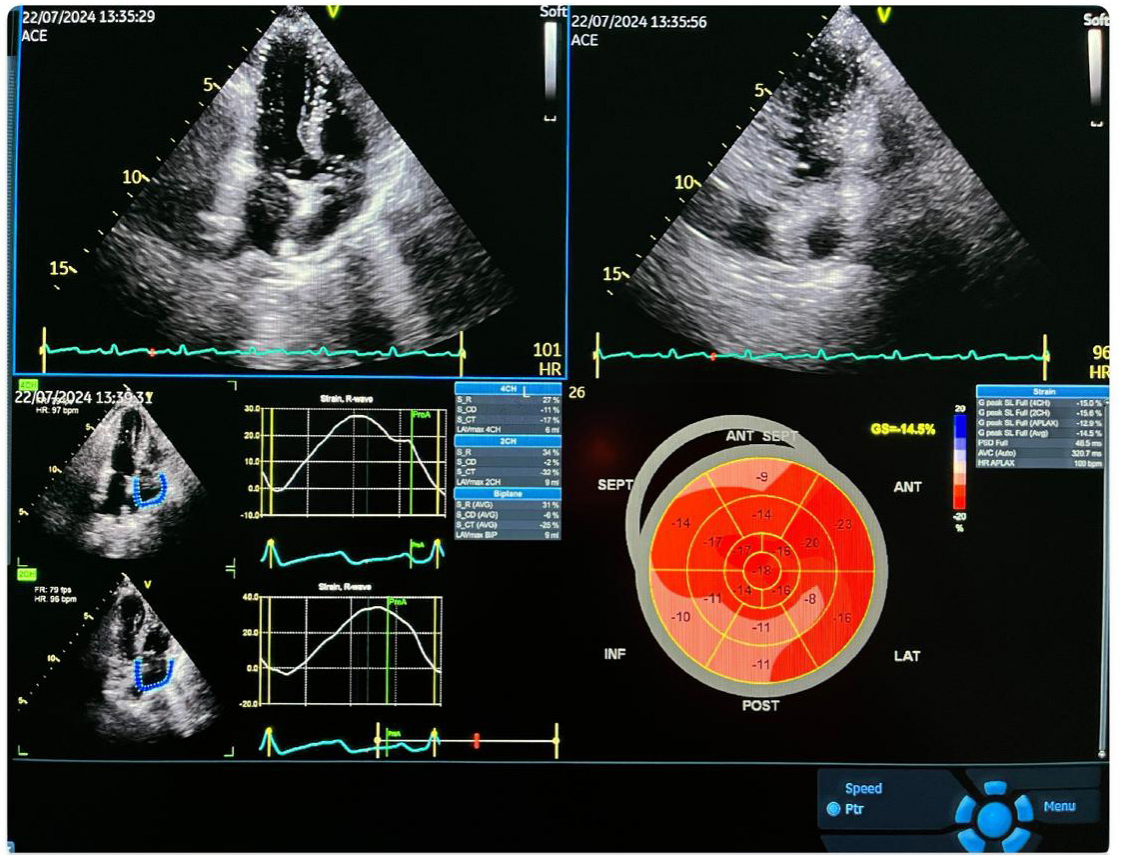
**A typical echocardiographic and global longitudinal strain measurement image**. ACE, angiotensin-converting enzyme; FR, frame rate; HR, heart rate; SEPT, septum; INF, inferior; ANT, anterior; LAT, lateral; POST, posterior; APLAX, apical long axis longitudinal strain; CH, chamber; AVG, average; LAV, left atrial volume; PSD, peak strain dispersion; AVC, aortic yalve clossure; S_R, strain reservoir (systole); S_CD, strain conduit early diastole; S_CT, strain contractile late diastole.

The left ventricular ejection fraction (LVEF) was calculated using the modified Simpson’s rule method [17]. Echocardiographic maximum left atrial volume was measured using the biplane area–length method from the apical four-chamber and two-chamber views at end-systole and was indexed to BSA (left atrial volume index, LAVI) [18]. Pulsed-wave Doppler was used to record the blood flow velocities at the transmitral inflow. The peak early (E) and late (A) filling velocities were recorded below the basal mitral annulus from the apical four-chamber views. The e’ and a’ waves were calculated using the basal septal and lateral segments in the left ventricle. Tissue Doppler velocities were measured from the apical four-chamber view at the mitral annulus septal and lateral basal segments. Signals were acquired over three end-expiratory cycles, and the average values were calculated for the early diastolic e’ velocities and systolic velocities. The E/e’ ratio was determined using the average e’ value from both sides of the mitral valve. Speckle tracking analyses were conducted using the device’s software program. Apical four-chamber, two-chamber, and three-chamber views were obtained. The left ventricle border was drawn automatically with the software program on the device; a manual correction was performed if required. Segments with unsatisfactory images were excluded from the evaluation. The global longitudinal strain (GLS) was determined by averaging the peak systolic strain values from 18 segments. As a result of processing the apical images, a 17-segment bull’s eye image was created. The device automatically measured left ventricular GLS values [19]. The longitudinal strain was found by dividing the shortening of the marked interval in systole by its original length, which was expressed as a percentage. The negative values indicated the shortening percentage (normal ranges: –15.9% to –22.1%) (Fig. 2) [19].

Left ventricular diastolic function was evaluated using four parameters: annular e’ velocity (septal e’ <7 cm/sec, lateral e’ <10 cm/sec), average E/e’ ratio >14, LAVI >34 mL/m^2^, and peak tricuspid regurgitation velocity >2.8 m/s [20]. The E/e’ ratio can be measured at the septal or lateral annulus, with typically higher velocities noted at the lateral annulus. However, this study used the average E/e’ ratio >14.

### 2.4 Statistical Analysis

Data were stored and analyzed using IBM-SPSS (IBM SPSS Statistics for Windows, Version 28.0.0, Armonk, NY, USA: IBM Corp.) statistical software. Levene’s test was used to examine the equality of variance (homogeneity). Continuous variables are presented as the median (minimum: maximum or interquartile range) or mean ± standard deviation values. Categorical variables are reported as n (%). According to the normality test results, the Mann–Whitney U or independent samples *t*-test was used to compare the two groups. Pearson Chi-square test, Fisher’s exact test, or Fisher–Freeman–Halton exact test were used to compare categorical variables. Multiple logistic regression analyses were conducted to identify the best independent predictors influencing the development of diastolic dysfunction in dialysis patients. Odds ratios (ORs), 95% confidence intervals (CIs), and Wald statistics were calculated for each independent variable. The Hosmer and Lemeshow goodness-of-fit test statistics, Cox and Snell R2, and Nagelkerke R2 were also obtained for each final model in the multivariate analyses. Pearson or Spearman’s correlation tests were used to analyze correlations between numerical variables. All statistical comparisons with a *p*-value below 0.05 were assumed to be statistically significant.

## 3. Results

A total of 47 patients who had been receiving dialysis treatment for an extended period were included in this study. In total, 23 of these patients received HD, and 24 received CAPD treatment. The characteristics of the HD and CAPD patient groups were compared. The mean age of the patients was 58.6 ± 11.2 years, and the mean dialysis time was 125.1 ± 35.2 months. A total of 36 (76.6%) patients were observed to have LVH, and 22 (46.8%) were observed to have diastolic dysfunction.

Table 1 compares HD and CAPD patient groups regarding baseline characteristics and dialysis parameters. When the baseline characteristics of patients receiving HD and CAPD were examined, it was found that the average age (*p*: 0.016) and BMI (*p*: 0.038) were significantly higher in the group receiving CAPD treatment. No significant difference was found between the groups when the patient’s comorbidities and the underlying primary cause of renal failure were considered. When the medication use of the patients was examined, it was observed that paricalcitol use was significantly higher in the HD group (*p*: 0.050). No significant difference was observed between the use of other drugs. When patients were evaluated according to dialysis parameters, residual urine was significantly higher in the group receiving CAPD (*p*: 0.049). Additionally, the calculated Kt/V ratio was significantly higher in the CAPD group (1.92 ± 0.39 *vs*. 1.63 ± 0.26, *p*: 0.005).

**Table 1.** **Baseline characteristics and dialysis parameters of all patients according to dialysis type**.

			CAPD (n = 24)	HD (N = 23)	*p*-value
Demographic characteristics					
	Age, y, mean ± SD		58.6 ± 11.2	49.4 ± 13.8	0.016
	Male sex, n (%)		10 (41.7)	16 (69.6)	0.080
	Weight, kg, mean ± SD		64.8 ± 9.9	64.1 ± 8.7	0.799
	Body mass index, kg/m^2^, mean ± SD		24.9 ± 3.07	22.9 ± 3.39	0.038
Primary disease					
	Unknown, n (%)		16 (66.7)	12 (52.2)	0.625
	Diabetic nephropathy, n (%)		3 (12.5)	5 (21.7)	
	Hypertensive nephropathy, n (%)		1 (4.2)	3 (13)	
	ADPKD, n (%)		2 (8.3)	2 (8.7)	
	Glomerulonephritis, n (%)		1 (4.2)	1 (4.3)	
	Pyelonephritis, n (%)		1 (4.2)	0	
Comorbidites					
	Hypertension, n (%)		15 (62.5)	16 (69.6)	0.609
	Diabetes mellitus, n (%)		2 (8.3)	3 (13)	0.666
	Hyperlipidemia, n (%)		11 (45.8)	6 (26.1)	0.159
	Obesity, n (%)		2 (8.3)	1 (4.3)	1.000
	Hepatitis B virüs, n (%)		1 (4.2)	1 (4.3)	1.000
Medications					
	Calcium channel blocker, n (%)		12 (50)	10 (43.5)	0.654
	B-blocker use, n (%)		9 (37.5)	5 (21.7)	0.341
	ACE inhibitor use, n (%)		15 (62.5)	12 (52.2)	0.474
	Cinacalcet, n (%)		4 (16.7)	8 (34.8)	0.193
	Paricalcitol, n (%)		0	4 (17.4)	0.050
	Sevelamer, n (%)		7 (29.2)	11 (47.8)	0.238
	Erythropoietin, n (%)		15 (62.5)	18 (78.3)	0.238
Dialysis parameters					
	Dialysis time, hours/month, mean ± SD		128.6 ± 37.4	121.5 ± 33.1	0.493
	Residuel urine, n (%)		10 (41.7)	3 (13)	0.049
	Daily urine amount, cc		275 (55–1500)	166 (100–200)	0.304
	Kt/V*, mean ± SD		1.92 ± 0.39	1.63 ± 0.26	0.005
	Dialysis access site, n (%)	Arteriovenous fistula	0	22 (95.7)	-
		Permanent dialysis catheter	0	1 (4.3)	
	Peritoneal transport rates, n (%)	High	3 (12.5)	0	-
		High average	12 (50)	0	
		Low average	8 (33.3)	0	
		Low	1 (4.2)	0	

Data are presented as median (interquartile range [IQR]), number (percentage), and mean ± SD of patients. Abbreviations: CAPD, continuous ambulatory peritoneal dialysis; HD, hemodialysis; ACE, angiotensin-converting enzyme; ADPKD, autosomal dominant polycystic kidney disease; SD, standard deviation. *Kt/V: K is the urea clearance, t is the time of dialysis, and V is the volume of distribution of patients.

Table 2 compares the CAPD and HD patient groups according to laboratory parameters. Albumin value was significantly lower in the CAPD group (33.9 ± 5.58 g/L *vs*. 39.8 ± 5.31 g/L, *p* < 0.001). Additionally, platelet (*p*: 0.018), calcium (*p*: 0.036), and low-density lipoprotein (LDL) cholesterol values (*p*: 0.005) were found to be significantly higher in the CAPD group. The glucose value was significantly lower in the CAPD group (*p*: 0.038). Regarding other laboratory parameters, no significant difference was detected between the two groups.

**Table 2.** **Laboratory parameters of all patients according to dialysis type**.

	CAPD (n = 24)	HD (N = 23)	*p*-value
Laboratory parameters			
Urea, mg/dL, mean ± SD	96.3 ± 31.2	113.6 ± 28.2	0.053
Creatinine, mg/dL, mean ± SD	7.89 ± 2.17	8.01 ± 1.95	0.848
Albumin, g/L, mean ± SD	33.9 ± 5.58	39.8 ± 5.31	<0.001
Hemoglobin, g/dL, mean ± SD	10.8 ± 2.3	10.9 ± 2.1	0.965
Lymphocyte, /mm^3^, mean ± SD	1.63 ± 0.39	1.32 ± 0.74	0.079
Platelet, /mm^3^, mean ± SD	237.4 ± 59.6	191.7 ± 67.3	0.018
hs-CRP (g/L), mean ± SD	37.8 ± 70.6	20.3 ± 36.2	0.566
Transferrin saturation, %, mean ± SD	29 ± 10.7	31.8 ± 16.5	0.496
Ferritin, mcg/L, mean ± SD	723 ± 807	923 ± 517	0.320
Calcium, mg/dL, mean ± SD	9.44 ± 0.88	8.91 ± 0.78	0.036
Phosporus, mg/dL, mean ± SD	4.75 ± 1.44	4.78 ± 1.21	0.927
Parathormone, pg/dL, mean ± SD	547.3 ± 647.9	677.1 ± 502	0.448
Glucose, mg/dL, mean ± SD	101 ± 26.7	126.5 ± 51.8	0.038
Total cholesterol, mg/dL, mean ± SD	173.3 ± 34.8	159.6 ± 33	0.173
LDL cholesterol, mg/dL, mean ± SD	144 ± 68.6	92.8 ± 37.2	0.005
Trygliceride, mg/dL, mean ± SD	140 ± 59.1	182.6 ± 136.3	0.169

Data are presented as mean ± SD of patients. Abbreviations: CAPD, continuous ambulatory peritoneal dialysis; HD, hemodialysis; hs-CRP, high-sensitivity C-reactive protein; LDL, low-density lipoprotein; SD, standard deviation.

Table 3 compares the CAPD and HD treatment groups according to echocardiographic parameters. When 2D echocardiographic parameters were examined, it was observed that LV mass value (260.7 ± 64 *vs*. 224 ± 59, *p*: 0.047) and LVMI value (153.3 ± 34 *vs*. 130.8 ± 38.5, *p*: 0.040) were significantly higher in the group receiving CAPD treatment. There was no notable difference between other 2D echocardiographic parameters. When tissue Doppler parameters were examined, the E/e’ ratio was calculated to be significantly higher in the CAPD group (*p*: 0.049). When GLS values were analyzed, the GLS values were significantly better in the group receiving HD treatment (*p*: 0.033). The patients were divided into four groups in terms of LVH (normal, mild, moderate, and severe). The number of patients with severe LVH was significantly more prevalent in the group receiving CAPD treatment (*p*: 0.002). Concentric hypertrophy was also significantly higher in the CAPD treatment group (*p*: 0.025). The echocardiogram of the patients with concentric hypertrophy under CAPD treatment is shown in Fig. 2.

**Table 3.** **Echocardiographic parameters of all patients according to dialysis type**.

			CAPD (n = 24)	HD (N = 23)	*p*-value
Two-dimensional echocardiographic parameters					
	LVDd, mm, mean ± SD		46.82 ± 3.61	45.03 ± 5.09	0.170
	LVDs, mm, mean ± SD		31.97 ± 6.09	30.89 ± 4.91	0.506
	IVSd, mm, mean ± SD		14.69 ± 2.74	13.70 ± 2.89	0.238
	PWd, mm, mean ± SD		12.82 ± 1.54	12.22 ± 1.94	0.244
	LV mass, g, mean ± SD		260.7 ± 64	224 ± 59	0.047
	LVMI, g/m^2^, mean ± SD		153.3 ± 34	130.8 ± 38.5	0.040
	RWT, mm, mean ± SD		0.550 ± 0.08	0.553 ± 0.14	0.921
	LA diameter, mm, mean ± SD		43.25 ± 5.1	44.56 ± 3.6	0.316
	LAVI, mL/m^2^, mean ± SD		32.2 ± 4.5	30.6 ± 4.2	0.222
	LVEF, %		55 (41.9–64.4)	51.2 (40–69)	0.296
Tissue Doppler parameters					
	LV transmitral E, cm/s, mean ± SD		84.77 ± 25.23	77.67 ± 34.51	0.424
	LV transmitral A (cm/s), mean ± SD		78.71 ± 27.02	72.82 ± 20.92	0.409
	E/A ratio, mean ± SD		1.19 ± 0.53	1.19 ± 0.53	0.932
	LV TDI septal S (cm/s), mean ± SD		7.49 ± 1.69	6.63 ± 1.59	0.083
	LV TDI septal E (cm/s), mean ± SD		5.94 ± 2.10	6.57 ± 1.92	0.259
	LV TDI septal A (cm/s), mean ± SD		9.59 ± 2.33	8.43 ± 2.05	0.077
	E/e’ ratio, mean ± SD		14.19 ± 3.80	12.38 ± 5.07	0.049
	GLS (%), mean ± SD		–15.55 ± 3.14	–17.51 ± 2.95	0.033
Left ventricular index and RWT values					
	Diastolic dysfunction, %		16 (66.7)	6 (26.1)	0.005
	LVH, %		22 (91.7)	14 (60.9)	0.002
	Left ventricular hypertrophy severity, %	Normal	2 (8.3)	9 (39.1)	0.002
		Mild	2 (8.3)	6 (26.8)	
		Moderate	2 (8.3)	3 (13)	
		Severe	18 (75)	5 (21.7)	
	Left ventricular geometry classification, %	Normal	0	2 (8.7)	0.025
		Concentric remodelling	2 (8.3)	7 (30.4)	
		Concentric hypertrophy	22 (91.7)	14 (60.9)	
		Eccentric hypertrophy	0	0	

Data are presented as number (percentage) and mean ± SD of patients. Abbreviations: CAPD, continuous ambulatory peritoneal dialysis; HD, hemodialysis; LVH, left ventricular hypertrophy; LVDd, left ventricular end-diastolic diameter; LVDs, left ventricular end-systolic diameter; IVSd, end-diastolic interventricular septum; PWd, end-diastolic posterior wall; LV, left ventricle; LVMI, left ventricle mass index; RWT, relative wall thickness; LA, left atrium; LAVI, left atrial volume index; BSA, body surface area; LVEF, left ventricular ejection fraction according to BSA; TDI, tissue Doppler imaging; GLS, global longitudinal strain.

The patients were categorized into two groups: those with diastolic dysfunction (22 patients) and those without (25 patients); the parameters that influenced diastolic dysfunction were evaluated. When the effects of age and BMI on the presence of diastolic dysfunction were assessed using univariate regression analysis, no significant effect was found (OR = 1.01, 95% CI: 0.96–1.07; *p*: 0.59 and OR = 1.04, 95% CI: 0.86–1.27 *p*: 0.67, respectively). Multivariate analysis showed that receiving CAPD treatment (OR = 90.48, 95% CI: 3.75–2180.57; *p*: 0.006), history of dyslipidemia (OR = 0.01, 95% CI: 0–0.33; *p*: 0.008), worse GLS (OR = 16.06, 95% CI: 1.30–198.64; *p*: 0.031), and calcium value (OR = 7.77, 95% CI: 1.37–44.12; *p*: 0.021) were independently associated with diastolic dysfunction.

In the average 6-month follow-up of the patient groups receiving CAPD and HD, no statistically significant difference was observed in the number of hospitalizations for various reasons (*p*: 0.341). During this follow-up period, seven patients in the CAPD group and five patients in the HD group died for multiple reasons, with no significant difference found in the number of deaths (*p*: 0.740).

## 4. Discussion

The results of this study demonstrated that LV diastolic dysfunction and deteriorations in left ventricular geometry were significantly higher in patients receiving long-term CAPD treatment than in patients receiving long-term HD treatment. CAPD treatment has advantages over HD, such as preserving residual kidney function, providing hemodynamic stability, and improving quality of life. To our knowledge, this is the first study in the literature to compare the association between left ventricular functions in patients undergoing long-term CAPD and HD treatments.

Although previous studies have described the development of LVH and diastolic dysfunction in patients with CKD and receiving HD treatment, this study examined the effects of HD and CAPD treatments on the development of LVH and diastolic dysfunction [8, 21]. The rate of diastolic dysfunction in the CAPD treatment group was significantly higher than in the HD treatment group (66.7% *vs*. 26.1%, *p*: 0.005). Additionally, the incidence of LVH was significantly higher in the CAPD treatment group compared to the HD treatment group (91.7% *vs*. 60.9%, *p*: 0.002). Based on these results, we can assert that cardiac dysfunctions, particularly LVH and diastolic dysfunction, occur significantly in patients undergoing long-term CAPD treatment.

LVH was present in 76.6% of the patients in our study. This rate was compatible with previous studies in the literature, which observed that the rate of LVH in CKD patients is between 70% and 85% [6, 22]. In our study, the rate of LVH was found to be high (91.7%), especially in the group receiving CAPD treatment. Subsequently, the analysis of previous study on this subject indicates that the incidence of LVH in patients receiving CAPD was approximately 75% [23]. This difference in LVH rate is because the average age of the patients included in this study was higher than that in other studies; moreover, the patients in this study had been receiving CAPD treatment for an extended period. Echocardiographically calculated LVMI values in the patient group receiving CAPD were significantly higher than in the HD group (*p*: 0.040). We can associate this result with the higher rate of LVH in the patient group receiving CAPD in our study. In the patient population in our study, the E/e’ ratio, one of the diastolic filling parameters, was found to be significantly higher in the CAPD group than in the HD group (*p*: 0.049). Consistent with our observation, another study found a higher E/e’ ratio in the CAPD group than in the HD group. However, the frequency of diastolic dysfunction was not specified in this cohort [24]. The GLS values of both groups in our study were below normal levels; the literature defines normal GLS values as >–18 [25]. The observed GLS values below this in our study can result from patients having CKD for a long time since previous data have shown that GLS decreases significantly in CKD patients [26]. When the between-group differences were compared in our patient population, the GLS values were significantly lower in the patients receiving CAPD treatment (–15.5 *vs*. –17.5, *p*: 0.033). We can attribute this result to the fact that the CAPD patients in our study had a higher rate of diastolic dysfunction and LVH count. When the relationship between GLS and mortality in CKD patients was previously examined in the literature, it was found that cardiac events and mortality were higher in patients with low GLS values [27]. Although it would be premature to formulate any conclusion due to the small number of patients in our study, we think future studies should investigate the effect of GLS on mortality in CAPD patients. When the drug use of the patients in our study was examined, it was found that paricalcitol use was higher in the HD group. We think this is because a previous study showed that, paricalcitol treatment, a vitamin D analog can treat hypercalcemia and hyperphosphatemia in patients receiving HD [28]. Interestingly, no prior studies have been conducted on this subject regarding CAPD patients.

When the dialysis parameters of the patients included in our study were examined, the number of patients with residual urine was significantly higher in the patient group receiving CAPD (*p*: 0.049). Recently it was shown that CAPD treatment protects residual renal functions better than HD treatment. Research has indicated that the residual urine volume in patients undergoing CAPD treatment is greater than in those receiving HD treatment [29]. The data we obtained in our study were observed to be compatible with the data in the literature. When evaluating the Kt/V ratio, one of the methods of measuring dialysis adequacy in the patient population in our study, we observed that this ratio is significantly higher in the CAPD group (1.92 *vs*. 1.63, *p*: 0.005). Although there are articles in the literature arguing that this Kt/V ratio used to measure dialysis adequacy should be >1.7, the HEMO study, one of the largest studies on this subject, showed that there is no significant difference in mortality and secondary outcomes between cutoff values of 1.7 and 1.3 [30]. With these findings, we showed that dialysis treatments were sufficient in both patient groups in our study, but patients in the CAPD group received more adequate dialysis.

When we examined the laboratory parameters of the patients included in our study, we found that albumin levels in the CAPD group were significantly lower than those in the HD group (*p* < 0.001). This result aligns with previous studies in the literature [31, 32]. Serum albumin is considered a biomarker of visceral protein and a key parameter for nutritional assessment [32]. One reason for the lower serum albumin levels in the CAPD group is thought to be the significantly lower protein intake, as indicated by the Semi-Semi-quantitative Food Frequency Questionnaire (FFQ), along with protein loss through the CAPD fluid [33]. Recently it was suggested that low serum albumin levels are more indicative of persistent inflammation and have limited value as a marker of nutritional status alone [34]. When the biochemical parameters of the patients were examined, it was determined that the calcium value was significantly higher in the patients receiving CAPD treatment, while the glucose value was higher in the patient group receiving HD treatment. However, the patients had no symptoms or findings related to these parameters. These differences in biochemical parameters were claimed to be due to the nutritional habits of the patients rather than the dialysis treatment they receive [35].

When the lipid profiles of our patients were examined, LDL cholesterol levels were significantly higher in the patients receiving CAPD (*p*: 0.005). High LDL cholesterol levels in the CAPD group were suggested to be due to the glycotoxic effects resulting from the glucose-based solutions used, which may indicate an increased risk of atherosclerosis [36]. Although improved survival rates have been observed in the first 3 years of patients receiving CAPD treatment, the benefits of long-term CAPD treatment remain controversial. Huang *et al*. [37] demonstrated that LDL cholesterol and apolipoprotein B levels were elevated in peritoneal dialysis patients and concluded that atherosclerosis may be more prevalent in this patient group [38].

### Study Limitations

The chief limitations of our single-center observational study were the modest patient sample size and the omission of some high-risk patient groups. Due to the small sample size, it is hard to generalize these results to all dialysis patients. While determining the patient population, patients who switched between dialysis methods were excluded from the study. Although this situation reduced the sample size, it can be shown as a factor that increased the power of the study since no switch between dialysis methods occurred. Peritoneal dialysis patients had a greater proportion of residual diuresis with higher Kt/V compared to hemodialysis patients. This contrasts with data in the literature, which show that better dialysis efficiency with residual diuresis has a lower impact on cardiac kinetics. Hemodialysis has been suggested to have a lower effect on cardiac kinetic functions than peritoneal dialysis; meanwhile, peritoneal dialysis has shown positive effects on cardiac ventricular capacity and heart failure management. Contrarily, peritoneal dialysis modality did not improve renal functions [39]. While interdialytic fluid retention was independently associated with mortality in hemodialysis patients, long-term HD and PD were not significantly different in terms of survival in end stage renal disease (ESRD) patients [40]. By including patients who were stable on dialysis modality in the long-term follow-up, we believe we have minimized the possibility of the dialysis method not being on optimal management, such as any fluid overload or blood pressure instability due to treatment inadequacy, toxicity, or suboptimal concentration of dialysis fluids. Therefore, the observed difference can be related to the dialysis method. Due to the lack of baseline and follow-up echocardiographic evaluations in our HD and CAPD patients, different confounding factors may have affected the echocardiographic findings at the end of such an extended period. The short follow-up period was a significant limitation in terms of prognosis.

## 5. Conclusions

The results of this study demonstrated that LV diastolic dysfunction and deteriorations in left ventricular geometry were significantly higher in patients receiving long-term CAPD treatment than in patients receiving long-term HD treatment. Despite adequate dialysis, more cardiovascular pathological changes were detected in patients receiving CAPD than for HD treatment. It would be advantageous to perform additional studies investigating the impact of these cardiovascular changes on prognosis and mortality over extended follow-up periods in larger patient cohorts, including those receiving CAPD treatment.

## Data Availability

The datasets used and/or analyzed during the current study are available from the corresponding author on reasonable request.

## Abbreviations

CVD, cardiovascular disease; ESKD, end-stage kidney disease; LVH, left ventricular hypertrophy; CKD, chronic kidney disease; HD, hemodialysis; CAPD, continuous ambulatory peritoneal dialysis; BMI, body mass index; AVF, arteriovenous fistula; URR, urea reduction ratio; TBW, total body water; TDI, tissue doppler imaging; LA, left atrium; IVSd, end-diastolic interventricular septum; PWd, end-diastolic posterior wall; LVDd, left ventricle end-diastolic diameter; LVDs, left ventricle end-systolic diameter; LVMI, left ventricular mass index; BSA, body surface area; RWT, relative wall thickness; LVEF, left ventricular ejection fraction; LAVI, left atrial volume index; GLS, global longitudinal strain.
